# EMDR therapy for PTSD after motor vehicle accidents: meta-analytic evidence for specific treatment

**DOI:** 10.3389/fnhum.2015.00213

**Published:** 2015-04-21

**Authors:** Maddalena Boccia, Laura Piccardi, Pierluigi Cordellieri, Cecilia Guariglia, Anna Maria Giannini

**Affiliations:** ^1^Department of Psychology, “Sapienza” University of RomeRome, Italy; ^2^Neuropsychology Unit, IRCCS Fondazione Santa Lucia of RomeRome, Italy; ^3^Department of Life, Health and Environmental Sciences, L’Aquila UniversityL’Aquila, Italy

**Keywords:** road traffic accident, ALE meta-analysis, acute psychological distress, traumatic single event, post-traumatic stress disorders

## Abstract

Motor vehicle accident (MVA) victims may suffer both acute and post-traumatic stress disorders (PTSD). With PTSD affecting social, interpersonal and occupational functioning, clinicians as well as the National Institute of Health are very interested in identifying the most effective psychological treatment to reduce PTSD. From research findings, eye movement desensitization and reprocessing (EMDR) therapy is considered as one of the effective treatment of PTSD. In this paper, we present the results of a meta-analysis of fMRI studies on PTSD after MVA through activation likelihood estimation. We found that PTSD following MVA is characterized by neural modifications in the anterior cingulate cortex (ACC), a cerebral structure involved in fear-conditioning mechanisms. Basing on previous findings in both humans and animals, which demonstrate that desensitization techniques and extinction protocols act on the limbic system, the effectiveness of EMDR and of cognitive behavioral therapies (CBT) may be related to the fact that during these therapies the ACC is stimulated by desensitization.

## Introduction

Traumatic events (including not only large-scale disasters but also common day-to-day events, such as Motor vehicle accidents (MVAs) are an important cause of psychological distress and psychiatric disorders. Harvey and Bryant (1998) reports the presence of acute stress disorders (ASD) in 13% of MVA survivors, and according to Mayou et al. (1993) one year after a MVA a quarter of those followed up showed psychiatric disorders, with 11% affected by post-traumatic stress disorders (PTSD).

PTSD is a relatively common psychiatric disorder occurring as a consequence of a major traumatic event. It is clinically characterized by the following symptoms: involuntarily re-experiencing phenomena (e.g., nightmares, flashbacks, intrusive images as well as recurrent distressing thoughts of the event); avoidance of talking about or being reminded of the traumatic event, negative alterations in thoughts and mood, emotional numbing and hyperarousal symptoms (e.g., sleep disturbance, difficulty in concentrating, increased irritability and hypervigilance) (DSM-IV; American Psychiatric Association, 2000, 2004).

Different types of psychological therapies have been proposed in the treatment of PTSD, including exposure therapy (Creamer et al., 2004), cognitive therapy (Resick and Schnicke, 1992; Ehlers et al., 2005), psychodynamic psychotherapy (Brom et al., 1989) and eye movement desensitization and reprocessing (EMDR; Shapiro, 1989). EMDR is currently an effective psychological treatment, recognized and recommended as a firstline treatment for trauma in numerous international guidelines (Bisson and Andrew, 2007). According to the review by Ponniah and Hollon (2009) EMDR ameliorates PTSD symptoms significantly more than waiting list, standard care, and pill placebo. They also reported that a number of studies found that EMDR was superior to trauma-focused cognitive behavioral therapies (CBT) on some measures of PTSD symptoms. However, all of these studies had mixed trauma samples. Another data coming from this review is that between 77 and 90% of EMDR patients no longer met diagnostic criteria for PTSD at the end of treatment. Ponniah and Hollon findings provide support for the use of EMDR for all patients with PTSD.

EMDR is a supplementary trauma-focused therapy that includes elements from other effective psychotherapies in a structured protocol drawn from an information processing model of PTSD (Bisson et al., 2013). It requires the individual suffering from PTSD to focus attention on a traumatic memory whilst simultaneously visually tracking the therapist’s finger as it moves across his/her visual field, and then to engage in a restructuring of the memory (Shapiro, 1995). Eye movements are the most common form of bilateral stimulation, but stimulation might also be auditory (alternating tones) or sensory (finger tapping). It acts by using dual attention tasks to help the patient process the traumatic event while focusing on negative trauma-related memories, emotions and thoughts during the performance of a task that requires a bilateral stimulation (e.g., eye movements; hand tapping; tones) until a growth in more positive trauma-related thoughts (Jensen, 1994; Shepherd et al., 2000; Marcus et al., 2004).

Since its discovery, EMDR has been considered one of the treatments of choice for PTSD, even though studies on its effectiveness are often hindered by methodological problems (see for a critical review Cahill et al., 1999), and in the view of some authors “what is effective in EMDR (imaginal exposure) is not new, and what is new (eye movements) is not effective” (McNally, 1999, p. 2). Although bilateral stimulation is discussed controversially (Cahill et al., 1999), growing evidence has demonstrated the effectiveness of EMDR in treating both PTSD in victims and mourning in survivors (Sprang, 2001; Solomon and Rando, 2007; see also meta-analysis studies: Bisson et al., 2013; Lee and Cuijpers, 2013; Watts et al., 2013). Lee and Cuijpers (2013) performed a meta-analysis in which 15 clinical and 11 experimental studies demonstrated different effects of bilateral stimulation through eye movements compared with those produced by other exposure therapies. Possible explanations for the effectiveness of alternating bilateral stimulation are: stimulation acts specifically on disintegrated information related to the traumatic event, or boosts the processing of emotionally memories or, last but not least, may enhance emotional processing in general (Sprang, 2001; Korn and Leeds, 2002). Herkt et al. (2014) recently observed in healthy subjects without post-traumatic symptoms increased activation in the right amygdala during alternating auditory bilateral stimulation, as used in EMDR, while processing emotionally negative stimuli. These authors suggested that the increase in limbic processing along with decreased frontal activation is in line with theoretical models (Shapiro, 1989, 2002) of how alternating bilateral stimulation might help with the therapeutic reintegration of information. Specifically, Shapiro (1989) suggests two possible interpretations for the effects of alternating bilateral stimulation: (i) it may boost the processing of any emotionally laden material in general; or (ii) it may have a specific effect just on disintegrated information related to the traumatic episode. Clinicians also observe a decrease in vividness and arousal related to trauma-associated stimuli after EMDR, and neuroimaging studies show that after EMDR there is a decreased activation in limbic areas and increased activation in prefrontal brain regions known to be responsible for cognitive control after the completion of successful treatments (Lansing et al., 2005; Pagani et al., 2007). Clinical trials suggest that different traumatic events interact with individual factors (such as personality, gender and genetic factors) and lead to different physical and behavioral outcomes as well as a different prevalence of PTSD (Ditlevsen and Elklit, 2012; Santiago et al., 2013; Husarewycz et al., 2014; Perrin et al., 2014). Even if altered brain areas after PTSD are common and play complementary roles in maintaining the PTSD symptomatology, such as fear conditioning of trauma-related stimuli and failing to recall fear extinction (Pitman et al., 2012). However, specific network of areas could be observed due to specific trauma. In details, PTSD after physical or sexual abuse modifies specific brain structures including the middle and anterior cingulate cortex (MCC; ACC), precuneus (pCU) and middle frontal gyrus (see Shin et al., 1999; Lanius et al., 2002, 2005). These brain regions are involved in pain processing, fear, sadness and proprioceptive information. Differently, in the PTSD after combat-related trauma alterations have been found in a network of areas including the bilateral insula, inferior frontal gyrus (IFG), posterior cingulate cortex (PCC), superior parietal lobe (SPL) and hippocampus (Pissiota et al., 2002; Britton et al., 2005; Geuze et al., 2007; Morey et al., 2008). Also these structures are known to be involved in emotional processing, especially of sadness (Vogt, 2005), and in monitoring internal body states, but they are also involved in a wide range of cognitive functions, including episodic memory, spatial navigation, imagining and planning for the future (Hassabis and Maguire, 2007; Vann et al., 2009; Boccia et al., 2014). A specific network of areas is present also in PTSD after catastrophe and includes the bilateral parahippocampal gyrus (PHG), right superior temporal gyrus (STG) and superior frontal gyrus (SFG; Hou et al., 2007; Chen et al., 2009; Mazza et al., 2012). Specifically, the PHG has a crucial role in spatial navigation and in scene perception (Epstein and Morgan, 2012) and it is reported only in this kind of trauma, likely due to the fact that the natural disasters mostly involved the surrounding environment and familiar places. Taking together these fMRI studies seem suggest that PTSD due to different kind of trauma can be different from a neurological and cognitive point of view. As a consequence also the variability in the psychological therapies effectiveness could be partially explained by the existence of different neural substrates underpinning the main disorder.

In view of this evidence, the main aim of the present study is to examine the extent to which neurobiological evidence supports the specific treatment of PTSD after MVA with EMDR. To pursue this aim we first reviewed previous neuroimaging studies about PTSD after MVA and those about neural correlates of EMDR. We hypothesized that by modulating the dysfunctional network of PTSD-MVA, EMDR may be the treatment of choice for patients who develop PTSD after a MVA. To test this hypothesis, we performed a meta-analysis of fMRI studies on PTSD after MVA to assess neural network functional changes in people suffering from PTSD following a MVA, using activation likelihood estimation (ALE; Eickhoff et al., 2009). Results have been discussed in light of current evidence about the neural underpinnings of EMDR, which suggests a specific and biologically-based approach to PTSD after MVA.

### Neural Correlates of PTSD After Motor Vehicle Accidents (MVA)

In the last decade different studies have assessed the neurobiological effect of PTSD after MVA (PTSD-MVA). fMRI studies mainly adopted trauma script-driven imagery paradigms, during which participants were required to remember olfactory, auditory, somato-sensory and visual sensations that were associated with their traumatic event (Frewen et al., 2008a,b; Osuch et al., 2008) or trauma-related pictures (Zhang et al., 2013). In PTSD-MVA patients, listening to trauma scripts leads to decreased activation in the bilateral amygdala and perirhinal cortex (Osuch et al., 2008). Studies also showed a negative correlation in PTSD patients between Emotional Awareness (measured by Levels of Emotional Awareness Scale, LEAS) and activation in the ACC during trauma script-driven imagery (Frewen et al., 2008a). In terms of emotional processing, the altered activation and deactivation in the limbic system (i.e., ACC, perirhinal cortex and amygdala) may be the biological substrates of emotional dysfunctions, such as emotional numbing and hyperarousal symptoms, observed in PTSD patients. Actually, Armony et al. (2005) found increased amygdala activation for unmasked happy faces, compared to fearful unmasked faces in PTSD patients. Their finding may contribute to explain why emotional numbing, that is the inability to fully experience positive emotions, is associated with PTSD (Litz and Gray, 2002). Furthermore, as stressed by Mazza et al. (2012) emotional numbing is closely associated with emotion processing, and points to a deficit in the acquired ability to understand/share emotions with others. fMRI evidence showed that people suffering from PTSD have functional alteration in emotional brain regions such as the amygdala and insula when presented with fearful and happy faces (Hendler et al., 2001; Armony et al., 2005).

In a delayed-response working memory task, where emotional (trauma-related) and non-emotional pictures were presented in the delay phase, PTSD-MVA patients showed higher activation in the emotion-processing regions, including the amygdala, precuneus and fusiform gyrus, but lower activation in the inferior frontal cortex, insula and left supramarginal gyrus than the control group (Zhang et al., 2013). The importance of these structures in emotional processing was demonstrated as far back in time as 1948, with the description of symptoms of Phineas Gage, a railroad construction foreman who as a consequence of a rock-blasting accident reported a large lesion in the left frontal lobe. His was one of the earliest documented cases providing evidence that frontal lobes were linked to judgment, decision-making, social conduct and personality (Bechara and Damasio, 2005) in spite of a preserved intellect. Not only do frontal lobes contribute to delineating personality and monitoring emotions, they also play a crucial role in emotional processing, performed by some limbic structures, such as the amygdala, cingulate gyrus, orbitofrontal cortex and enthorinal cortex. Specifically, the amygdala is involved in fear conditioning (for a review see Janak and Tye, 2015).

In addition, studies using resting-state fMRI paradigms demonstrated that individuals with PTSD-MVA showed a different amplitude of low-frequency fluctuation values (fractional amplitude of low frequency fluctuation, ALFF), with an increased ALFF in the left medial prefrontal cortex and the right ACC (Bing et al., 2013). A PET study also found a hyperperfusion in the right medial prefrontal cortex and ACC in people with PTSD-MVA (Osuch et al., 2008). Furthermore, Qin et al. (2012), using resting-state fMRI to assess functional connectivity in individuals with PTSD-MVA, found that compared with control participants people with PTSD exhibited decreased PCC connectivity with the right lingual and middle temporal gyri, as well as with the left lingual gyrus. On the other hand, the left inferior temporal gyrus, right middle temporal gyrus, left middle temporal gyrus and insula, left medial frontal lobe and ACC, and right medial frontal gyrus showed increased PCC connectivity in people with PTSD (Qin et al., 2012). These results suggest that patients with PTSD showed different patterns of resting-state functional connectivity. This is a method of functional neuroimaging that makes it possible to evaluate brain region interaction while individuals are not performing any cognitive task. Specifically, Qin et al. (2012) suggested that the increase in functional connectivity could explain the abnormal emotional responses in patients with PTSD, and interpreted the decrease in functional connectivity as being due to comorbid psychiatric disorders.

Structural studies of PTSD after MVA mainly found a decrease in cortical thickness in the left medial prefrontal cortex, ACC and middle frontal gyrus as well as in the right STG (Bing et al., 2013). They also showed increased ALFF values in the prefrontal cortex, ACC and cerebellum. This increased regional activity is known to be important for emotional processing (for an extensive review see Drevets, 2000).

Furthermore, structural connectivity network analysis in people with PTSD-MVA, assessed by using diffusion tensor tractography, demonstrated abnormal global properties mainly due to an increased shortest path length (Long et al., 2013). Furthermore, individuals with PTSD-MVA showed enhanced nodal centralities in the bilateral anterior cingulate and pallidum, and in the hippocampus and PHG, and decreased nodal centralities in medial orbito-frontal cortex (Long et al., 2013). These authors suggest that as the orbitofrontal cortex is involved in the extinction of conditioned fear, the disruption of the nodal centralities of the orbitofrontal cortex plays a role in the persistence of PTSD symptoms.

In summary, fMRI evidence showed that PTSD-MVA is characterized by the alteration in the limbic system (i.e., ACC, perirhinal cortex and amygdala) in the left medial prefrontal cortex, in the orbitofrontal cortex and in the middle frontal gyrus as well as in the right STG. These areas are also involved in different degree of changes also in PTSD caused by other traumas, however, the limbic system and in particular of the ACC are more involved in PTSD due to MVA with respect to other traumas. However, more fMRI studies should be performed to better understand neural mechanisms underlying different type of traumas and above all to state that an area is involved only in one type of trauma with respect to another one it should be performed a meta-analysis in which neural correlates coming from different studies on different traumas are analyzed with the aim to individuate cluster of activations.

### Neural Correlates of EMDR

While several studies have assessed the behavioral effect of EMDR therapy (as reported above), less attention has been given to neural correlates of recovery after EMDR. One of the most important findings comes from neurophysiological studies, using electroencephalography (EEG). It has recently been found that EMDR facilitates the processing of traumatic memories in eye movement desensitization by improving interhemispheric coherence, measured by EEG (Samara et al., 2011). With regard to brain regions supporting EMDR efficacy in PTSD, both functional and structural neuroimaging studies have been conducted. In a single case analysis Levin and colleagues, using Single Photon Emission Computed Tomography (SPECT), found that recovery from PTSD symptoms after EMDR is correlated to increased activation in the anterior cingulate gyrus and the left frontal lobe (Levin et al., 1999). These authors concluded that successful treatment of PTSD does not reduce arousal at the limbic level, but rather enhances the ability to differentiate real from imagined threats (Levin et al., 1999). Further studies shed some light on the possible neural mechanism involved in recovery from PTSD after EMDR therapy. Lansing et al. (2005), using SPECT to assess the neural effect of EMDR therapy in Police Officers with PTSD, found decreased activation in the left and right occipital lobe, left parietal lobe, and right precentral frontal lobe and increased activation in the left IFG, after 10 h (4 sessions) of EMDR therapy. Furthermore, Nardo et al. (2010) used MRI and Voxel Based Morphometry to assess neural predictors of EMDR therapy in people developing PTSD after occupational trauma. These authors found that individuals who did not respond to EMDR showed a significantly lower gray matter density than responders in the bilateral posterior cingulate, as well as in the right anterior insula, anterior PHG and amygdala. They also found that gray matter density negatively correlated with trauma load in the bilateral posterior cingulate, left anterior insula, and right anterior PHG. Thus, they concluded that gray matter lower density in limbic and paralimbic cortices was associated with PTSD diagnosis, trauma load and EMDR treatment outcome.

As regards the neurobiological mechanism underlying EMDR effects, it has been proposed that the repetitive redirecting of attention in EMDR induces a neurobiological state, similar to that of REM sleep, which allows for re-processing of traumatic memories (Stickgold, 2002). Specifically, Stickgold (2002) proposed that EMDR leads to a reduction in the strength of hippocampally mediated episodic memories and amygdala-dependent emotional memories. This hypothesis is consistent with the neuroanatomical findings reported above.

The above-reported neuroimaging evidence seems to suggest that by modulating the neural circuits altered in PTSD after MVA, EMDR may represent the “treatment of choice” for PTSD-MVA. EMDR modulating activation in limbic areas and prefrontal regions (Lansing et al., 2005; Pagani et al., 2007) may actually result in the regulation of the dysfunctional network in PTSD-MVA patients, allowing for the reprocessing of episodic and emotional traumatic memories.

In the paragraphs below we will present results from a meta-analysis of the previous neuroimaging studies of PTSD-MVA, which suggest a biological-founded model for treating PTSD after MVA.

## Meta-Analysis

### Studies Selection

A systematic method was adopted to review literature. The search was carried out using PubMed, a free digital archive of biomedical and life sciences journal literature in which all articles using fMRI method are reported. Relevant articles were identified through searches using the following string: (PTSD FMRI) OR PTSD PET. Searches of this database were limited to articles written in English. This produced 615 papers. Our a-priori inclusion criteria for papers were: (1) Inclusion of whole-brain analysis performed using magnetic resonance imaging (MRI) or positron emission tomography (PET); this excluded papers that reported only results from ROI analysis. (2) Providing of coordinates of activation foci, either in Montreal Neurological Institute (MNI) or Talairach reference space. (3) All participants in the studies were diagnosed with PTSD after MVA. (4) PTSD was diagnosed according to the DSM-IV diagnostic criteria, including symptoms persisting for 6 months or more. (5) Other psychiatric disorders or other trauma-related PTSD were excluded. (6) Only group studies were included. Two authors (L.P. and M.B.) independently identified studies matching with the criteria reported above. Only published studies were included in our meta-analysis.

Using these criteria, we selected only 6 papers reporting 9 individual experiments on PTSD-MVA patients (440 subjects; 30 activation foci). Meta-analysis was carried out on selected studies using the “activation likelihood estimation” (ALE) analysis. Studies are summarized in Table 1.

**Table 1:** **fMRI Studies on PTSD-MVA included in the ALE meta-analysis**.

Paper	N	Studies	Time since MVA
Bing et al., 2013	40	1	7.2 ± 1.6 months
Frewen et al. (2008a)	41	1	6.11 ± 9.61 years
Frewen et al. (2008b)	26	2	-
Osuch et al. (2008)	62	3	-
Qin et al. (2012)	81	1	6 months
Zhang et al. (2013)	40	1	-

*Notes. DSM-IV diagnostic criteria were adopted in the three studies in which time is not specified*.

### Activation Likelihood Estimation

Activation likelihood estimation (ALE) analyzes the probability that a voxel will contain at least one of the activation foci; it is calculated at each voxel, and results in a thresholded ALE map. ALE assesses the overlap between foci by modeling the probability distributions centered at the coordinates of each one (Eickhoff et al., 2009).

We performed a general ALE analysis to determine whether a consistent neural substrate of PTSD was found across neuroimaging studies of PTSD after MVA. Then, by means of meta-analytic connectivity modeling (MACM), used to assess the functional connectivity of specific brain regions (Robinson et al., 2010), we investigated the functional connectivity of a brain region that emerged as showing a consistent modification in PTSD-MVA, from the first ALE analysis.

The ALE meta-analysis was performed using GingerALE 2.1.1 (brainmap.org) with MNI coordinates (Talairach coordinates were automatically converted into MNI coordinates by GingerALE). According to Eickhoff et al.’s (2009) modified procedure, the ALE values of each voxel in the brain were computed, and a test was performed to determine the null distribution of the ALE statistic of each voxel. The Full-Width Half-Maximum (FWHM) value was automatically computed, as this parameter is empirically determined (Eickhoff et al., 2009). The thresholded ALE map was computed using *p* values from the previous step and a False Discovery Rate (FDR) at the 0.05 level of significance (Tom Nichol’s FDR algorithm). Moreover, a minimum cluster size of 200 mm^3^ was chosen. A cluster analysis was performed on the thresholded map. The ALE results were registered on an MNI-normalized template (brainmap.org) using Mricro.^1^

## Results

**Figure 1:**
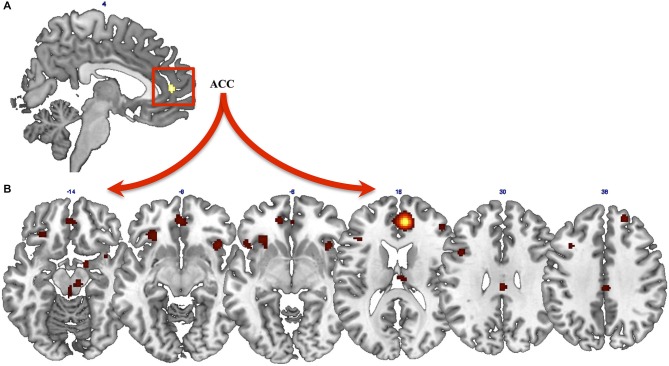
**Results from ALE meta-analysis. (A)** Results from the general ALE meta-analysis on neural modifications of PTSD after Motor vehicle accidents (MVAs). **(B)** Functional connectivity of the anterior cingulate cortex (ACC), assessed by means of meta-analytic connectivity modeling (MACM) analysis.

Results from the general ALE meta-analysis showed a cluster of activation in the right ACC (*x* = 8; *y* = 46; *z* = 12) (Figure 1A). Thus, we assessed the functional connectivity of the ACC by means of MACM. MACM showed patterns of functional connectivity of the ACC, especially at the level of the frontal, parietal and limbic lobes (Figure 1B). In detail, we found clusters of activation in the bilateral ACC, insula, IFG, left precentral and cingulate gyri and claustrum, as well as in the right middle and superior frontal gyri, inferior parietal lobule, globus pallidus and thalamus (Figure 1B).

## Discussion

The main aim of the present study is to provide neurobiological evidence on which to base a biological-founded treatment model for PTSD after MVA. Neuroimaging evidence reviewed in the introduction section appears to suggest that by modulating the neural circuits altered in PTSD after MVA, EMDR may represent a “treatment of choice” for PTSD-MVA. Thus, we performed a comprehensive meta-analysis of neuroimaging studies of PTSD-MVA to assess (a) brain areas showing consistent modifications in PTSD after MVA; and (b) their functional connectivity.

Global results from the present meta-analysis demonstrate that PTSD-MVA is correlated with consistent neural modifications mainly located at the level of the right ACC, whose activity is in turn strongly correlated with activity in the frontal, parietal and limbic lobes. Also based on previous neuroimaging evidence of neural underpinnings of EMDR (Levin et al., 1999; Lansing et al., 2005; Nardo et al., 2010; Samara et al., 2011), we propose that by modulating the dysfunctional network of PTSD-MVA, EMDR may be a treatment of choice for patients who develop PTSD after a MVA. In other words, by working on the neural network that we found altered in PTSD-MVA, EMDR may produce a normalization of the dysfunctional network.

The neural impact of other therapies does in any case need to be considered. Firstly, the main element of all psychological therapies treating anxiety disorders (including PTSD) is exposure to the feared object or context (Joseph and Gray, 2008). Exposure is based on extinction learning (Myers and Davis, 2007; Quirk and Mueller, 2008; Herry et al., 2010), which relies on inhibition of the amygdala by the prefrontal regions and engages executive functions, mainly recruiting the dorsolateral prefrontal cortex (LeDoux, 2002). Furthermore, the greater activity in the left frontostriatal circuit is associated with lower PTSD symptom severity after treatment with CBT (Falconer et al., 2013). Also, the greater bilateral amygdala and ventral anterior cingulate activation is associated with a poor outcome after CBT (Bryant et al., 2008). CBT has been found to regulate brain activations at the level of frontal network and of the limbic structures (Goldapple et al., 2004). In fMRI studies the changes after psychotherapies administered to face major depression, phobia, obsessive compulsive disorders emerge that the psychotherapies (i.e., CBT, Interpersonal Therapy, Behavioral Therapy; Mindfulness based cognitive therapy; computer assisted cognitive remediation and psychodynamic therapy) mainly targets at brain networks (including precuneus and orbito frontal cortex) able to mediate a top-down effect on symptoms improving. They might induce modifications especially in the medial portion of the parietal (i.e., precuneus, as described before), frontal (i.e., superior and inferior frontal gyri) and temporal lobes (i.e., middle temporal gyrus) (Brody et al., 2001; Yamanishi et al., 2009; Buchheim et al., 2012; Ives-Deliperi et al., 2013; Meusel et al., 2013; Yoshimura et al., 2014). Some brain areas modulated by psychotherapies are common with those modulated by EMDR, such as frontal, parietal and limbic structures. However, EMDR acts on ACC that is an important role in the fear extinction. ACC is also a structure modified in PTSD and specifically in PTSD due to MVA.

A systematic review of psychological therapies for chronic PTSD showed that the severity of PTSD symptoms is reduced by using individual trauma-focused cognitive behavioral therapy (TFCBT) and EMDR. This result emerged from 70 studies involving a total of 4,761 participants, demonstrating more effective TFCBT and EMDR than waitlist/usual care (Bisson et al., 2013). These authors found no statistically significant difference between individual TFCBT, EMDR and Stress Management (SM) immediately post-treatment, although there was some evidence that individual TFCBT and EMDR were superior to non-TFCBT at follow-up, and that individual TFCBT, EMDR and non-TFCBT were more effective than other therapies. Non-TFCBT was more effective than waitlist/usual care and other therapies. Worthy of note however is the fact that a considerable proportion of patients (30–50%) do not respond to TFCBT (Bradley et al., 2005). Generally speaking, both TFCBT and EMDR are highly efficacious in reducing PTSD symptoms, due to common underlying factors involved in different treatments for PTSD, such as an exposure model to treat the trauma, which causes the patient to rethink previous interpretations of the traumatic event, addressing faulty cognitions or maladaptive copying strategies, and also promoting fear extinction (Bradley et al., 2005).

EMDR on the other hand has repeatedly been found to rely on the ACC (Yamasaki et al., 2002), which we found to be biased in PTSD after MVA. Thus, EMDR may be considered one of the treatments of choice for PTSD after MVA, since it directly acts on the brain region showing an alteration in these patients. It is also possible that the effectiveness of EMDR on PTSD-MVA is related to its action on conditioned fear, mediated by ACC, due to repeated exposure to the traumatic event, while other cognitive therapies like CBT, promoting the rationalization and acceptance of the trauma, modulate the response of the prefrontal cortex, inducing a secondary response on the ACC. This different action could explain the difference between EMDR and CBT, supporting Solomon and Rando (2012), who reported positive treatment effects, obtained more quickly with EMDR compared with other forms of therapies.

Anyway, EMDR cannot be considered suitable just for MVA-PTSD. Actually, EMDR has been successfully used, to treat PTSD after different traumatic events (Lansing et al., 2005; El Khoury-Malhame et al., 2011; Bisson et al., 2013). Unfortunately, as highlighted in the introduction, the paucity of studies assessing neural correlates of EMDR therapy does not allow us to come to any definite conclusion in respect of firstline treatment, even if current data support a biological foundation for EMDR in treating PTSD-MVA and PTSD after other types of traumatic events, such as rape, work-related accidents or duty-induced PTSD (Lansing et al., 2005; El Khoury-Malhame et al., 2011; Bisson et al., 2013). It should be noted that current literature lacks systematic studies that take into account the nature of the trauma event and the treatment effect. Thus, even though further systematic studies are needed to prove that EMDR is one of the best approaches to treating PTSD following a MVA, we believe it may be one of the most effective.

## Conflict of Interest Statement

The authors declare that the research was conducted in the absence of any commercial or financial relationships that could be construed as a potential conflict of interest.

## Footnotes

^1^http://www.mccauslandcenter.sc.edu/mricro/index.html
